# Cultural significance of the flora of a tropical dry forest in the Doche vereda (Villavieja, Huila, Colombia)

**DOI:** 10.1186/s13002-018-0220-0

**Published:** 2018-03-22

**Authors:** Jeison Herley Rosero-Toro, Luz Piedad Romero-Duque, Dídac Santos-Fita, Felipe Ruan-Soto

**Affiliations:** 1 Universidad de Ciencias Aplicadas y Ambientales, 222 St. 55-37, E-111166 Bogotá, Colombia; 2 Asociación Etnobiológica Mexicana A.C., Calle Profesor Felipe W. Mijangos, Colonia 12 de Junio, E-29243 San Cristóbal de Las Casas, Chiapas Mexico; 3 Centro de Investigaciones Multidisciplinarias sobre Chiapas y la Frontera Sur, UNAM, Calle María Adelina Flores 34-A, Barrio Guadalupe, CP 29230 San Cristóbal de Las Casas, Chiapas Mexico

**Keywords:** Ethnobotany, Cultural significance, Use and management, Tropical dry forest, Colombia

## Abstract

**Background:**

In Colombia, ethnobotanical studies regarding plant cultural significance (CS) in tropical dry forests are scarce and mainly focused on the Caribbean region. Different authors have indicated that the plants with the most uses are those of greater cultural importance. Additionally, gender differences in knowledge and interest in natural resources has been widely recorded. This study evaluated the cultural significance of plants in the Doche community, in the Department of Huila. Furthermore, it evaluates the richness of plant knowledge among local inhabitants, looking for testing the hypothesis that the CS of plants positively correlates to the number of uses people inform about, and that there are significant differences on the richness of ethnobotanical knowledge between men and women in this community.

**Methods:**

The ethnobotanical categories: “food,” “condiment,” “economy,” “fodder,” “firewood,” “timber”, “medicine,” and “others” were established to carry out semi-structured interviews, social cartography, and ethnobotanical walks. The frequency of mention was calculated as a measure of CS. The richness of knowledge of each collaborator was obtained. Non-parametric tests were performed to determine whether differences between the numbers of mentioned species existed between genders and ethnobotanical categories. Finally, Pearson correlation tests determined the relationship between CS and the number of ethnobotanical categories.

**Results:**

A hundred useful species were registered in crops and forests. The most abundant categories were medicinal (45 species), firewood (30), and fodder (28). The most culturally significant species according to frequency of mention were *Pseudosamanea guachapele*, *Guazuma ulmifolia*, *Manihot esculenta*, and *Musa balbisiana*. The species with the most registered uses (five) were *Guazuma ulmifolia* and *Gliricidia sepium*. We found a correlation between CS and the number of uses per ethnobotanical category, but no significant difference between genders regarding ethnobotanical knowledge.

**Conclusion:**

Frequency of mention provides relevant information about the CS of species. Furthermore, it aids to establish sustainable use of tropical dry forests without loss of resources parting from strategies designed from within the Doche community and based on their ethnobotanical knowledge. We found that the number of uses of a plant is correlated with its degree of cultural importance. On the other hand, no significant differences were found between genders regarding ethnobotanical knowledge; that is, both men and women have similar roles in the community, which allows them to recognize the same uses per species.

## Background

The services provided by ecosystems are valued differently by different actors according to socio-ecological contexts and cultural and economic interests [1, 2]. The measure of this value can be ecologic, economic, and socio-cultural [3]. This last category has become a relevant tool to learn of the significance and the benefits that ecosystems provide to human communities, resulting in a combination of social perception and the capacity of an ecosystem to satisfy the needs of human groups [4–6]. While this information does not necessarily mean a monetary estimation of the services, it does reflect the relevance of the services provided [4]. Thus, cultural significance can be a valuable tool for such purposes of evaluating ecosystem services [7].

The cultural significance of a species has been defined by Hunn [8] as the value or role it has within a particular community; this includes species with high and low relevance for a social group and it may vary according to use and appreciation of a species by people [9, 10]. Research on cultural significance has used for different research approaches, using different methods: participant techniques, group interviews, community workshops, participant observation, academic and community group opinions, and others [11, 12]. From quantitative ethnobotany, numerous ways to evaluate the significance of a particular taxon have been proposed, the most popular of which are those based on informant consensus [13, 14]. The most popular indicators in these cases include frequency of mention [15].

Different ethnobotanical studies have speculated about the features that plant species that are considered highly culturally significant must share. Aspects such as availability [16–18], features of their biological cycle [19], or other specific features such as biomass and size [20], to mention some few, have been explored as factors that might explain cultural significance. For some researchers, the more uses a plant has (either as food, construction material, medicine, religious objects, or any other) the greater its degree of cultural significance is [16, 21, 22]. Therefore, the sum of uses is a very widespread method, which allows to quickly quantify the importance of species [22].

On the other hand, indexes like the Knowledge Richness Index have been used to evaluate the degree of knowledge a user has about the possibilities of their useful flora and whether significant differences exist in the knowledge of different socio-demographic sectors [23, 24], given that clearly different social groups have different roles that could affect the amount and quality of knowledge of useful flora [24–26]. For gender, different authors have reported that preferences on plant species, as well as the general interest in natural resources, may be different in men and women and, therefore, both have different priorities in the management of natural resources [27]. Particularly from ecofeminism, it is postulated that women, through their daily activities, have a more intense bond with their environment, which makes them carriers of a special interest in the conservation of nature and gives them extensive knowledge about the natural resources that surround their communities [28].

In Colombia, ethnobotanical studies have dealt with inventories of useful flora [29–31] and agro-biodiversity in traditional production systems [32, 33]. Studies of ethnobotany in tropical dry forests are scarce and have been focused on the Caribbean region, mainly reporting inventories of uses and vernacular names for useful plants [34–36]. Thus far, research in the department of Huila has been mainly centered in analyzing the functional and nutritional properties of Passifloraceae [37], as well as identifying the non-timber forest resources with the greatest potential for medicine and commerce in the mid and lower basins of Las Ceibas river [38]. In the Doche vereda, where this study was carried out, there is around 35.3% of tropical dry forest left [39]. The strong pressure that is put on this ecosystem through timber, fodder, and firewood extraction along with the different agricultural activities supporting economic and subsistence activities have caused the ecosystem services to be less available and accessible to the community. Furthermore, no studies have evaluated the most culturally significant species in this community, the features these plants share, or whether knowledge differs between men and women.

In this context the following questions arise: What are the used species in the Doche community with the greater cultural significance (CS)? Can the number of uses a plant has be a factor that explains its CS? And, is the richness of knowledge about these species different for men and women? This study aims to determine the CS of different used plants in the Doche community in the Huila Department in Colombia. Furthermore, it evaluates the richness of plant knowledge among its inhabitants, testing the hypothesis that the CS of plants positively correlates to the number of uses they are given and that there are significant differences on the richness of ethnobotanical knowledge between men and women in this community.

## Methods

### Study area

**Fig. 1:**
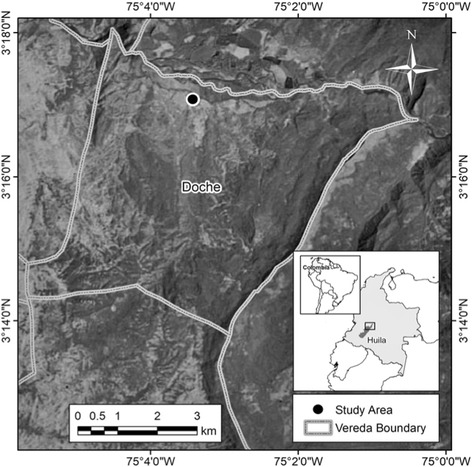
Location of Doche vereda (Villavieja, Huila, Colombia). Image by Trejo-Rangel (2017)

This study was carried out in the Doche vereda, old Doche Hacienda located in the eastern portion of the municipality of Villavieja (Huila, Colombia) located at 3° 17′ 5.07″ North and 75° 3' 25.11″ West (Fig. 1), with an extent of 3870.6 ha [40]. The area has a transitional climate spanning from warm-dry to warm-very dry; the mean monthly temperature is 28 °C with scarce rains. The vegetation is that of a tropical dry forest, where average annual temperature is ≥ 25 °C, annual rains span from 700 to 2000 mm, and there are three or more dry months during the year (rain < 100 mm/month) [41].

Fandiño and Wyngaarden [40] registered a population of 79 inhabitants, and according to the Development Plan for Villavieja [42], 18 residences can be found there. According to estimations in the field, there are around 60 people currently living in Doche. Although agriculture and stockbreeding have grown following the creation of an irrigation system, these activities have decreased. Because of this situation, people have migrated to other regions within this department. The residents of this area depend on the sale of rice, bananas, sweet potato, and cocoa, and, to a lesser degree, on goat and sheep stockbreeding for family sustenance.

### Data collection and analysis

Between December 2015 and August 2016, we worked with 18 people (seven men and 11 women) belonging to 12 families. The community authorized the use of all data obtained via the proposed methodologies through previous, free, and informed consent. The participants were selected following three criteria: (a) being current residents of Doche, (b) carrying out activities related to natural resource use in the study area, and (c) having time availability to participate in the project.

To register the cultural value of useful flora in the tropical dry forest, eight ethnobotanical categories were established; these were “food” (cultivated and wild edible species), “condiment” (species used as additional ingredients to prepare food), “economic” (species generating an income by being sold), “fodder” (species used as livestock food), “firewood” (species used for heating to cook food), “timber” (species used for construction to make beams, fences, or other things), “medicine” (species that prevent or cure ailments in humans), and “others” (useful species that are not included in the aforementioned categories). Based on these categories, the semi-structured interviews were carried out [43] among participants to recognize species of cultural significance.

To establish the cultural significance (CS) of plant species, those interviewed listed the most important species in each ethnobotanical category and their frequency of being mentioned was calculated [44–46] by adding the number of times each species was mentioned [47, 48].

Additionally, the Knowledge Richness Index (RQZ) was calculated to estimate the richness in the knowledge of each person about the uses of plants in their region [23]. For accomplishing such purpose, the following equation was used:

$$ \mathrm{RQZ}=\frac{\mathrm{EU}}{\ \mathrm{maximum}\ \mathrm{EU}\ \mathrm{value}} $$

In this equation, EU = the number of useful species to make up for services reported by a participant and maximum EU value = total number of useful species needed to make up for reported services in the region by all participants. The value of this index varies between zero and one, where one represents the maximum knowledge of useful plants in the region.

The results from the interviews were analyzed with non-parametric *U* Mann-Whitney tests [49] to determine whether differences exist in the number of mentioned species between genders and between ethnobotanical categories. Likewise, Pearson correlations were calculated to determine whether a greater CS (obtained through the frequency of mention) would include the species included in the greater number of ethnobotanical categories. These statistical analyses were performed using Minitab 16. Additionally, a Principal Coordinates Analysis (PCO) was carried out to determine the similarity of the reported useful flora between interviewed people and ethnobotanical categories. This analysis was carried out using the 2.11 version of the Numerical Taxonomy and Multivariate Analysis System (NTSYSPC) software [50].

Finally, a social cartography workshop was carried out [51] to find the areas from which plant species are extracted. To recognize the known vegetation’s vernacular names, ethnobotanical walks were taken in the usage areas in order to identify useful species [51]. Plant material that was not identified in the field was collected and registered in photography for botanical backup. The determination of plants was carried out in the biological collection at the Universidad de Ciencias Aplicadas y Ambientales (UDCA) in Bogota, using the TROPICOS platform to corroborate current scientific names. These specimens were not included in the biological collection due to their lack of minimal required features, such as flowers and/or fruits.

## Results

**Fig. 2:**
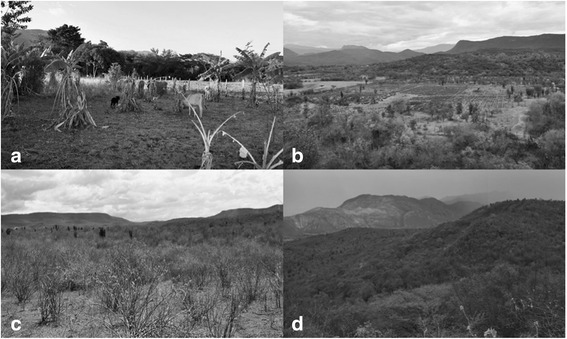
Areas in which vegetable species are used in the Doche community (Villavieja, Huila), both croplands and forests. **a** Location of the cropland along the banks of the Cabrera river and **b** in the tree-covered Savannah. The forest area is located between the **c** tree-covered Savannah and **d** Cerro Saltarén (photos by J. H. Rosero-Toro)

We found that the areas in which species are used by the Doche community are croplands and cattle areas up to a hectare in extension. These are located in tree-covered savannah and the banks of Cabrera river (Fig. 2a, b), and the wooded area spanning from the tree-covered savannah to the conservation area called Cerro Saltarén, where logging and herding activities take place (Fig. 2c, d). In croplands, 58 plant species are used, while 34 species come from forests; eight species can be found in both environments (Appendix).

Regarding the access to utilized flora, it is currently allowed to log only two individuals of wild timber species per family each year, while no limitations are set for firewood species. Approximately 75% of the families interviewed extract timber once or twice a week, while 25% of them seek this resource three or four times a week. Fodder material is extracted daily by 35% of the families, while 47% of them extract it once or twice a week, and 18% only three to four time a week. Contrastingly, the interviewed population showed no knowledge of the magnitude of use of species in the food, condiment, economy, medicine, and others categories.

In all, 100 species of used flora were recorded. These belong in 94 genera and 39 families; four species were not taxonomically identified since they were not found in the study area (see Appendix). The most used family was Fabaceae, which included 13 genera and 15 used species, while 24 families included only one used species each. The *timber* category groups 20 species. From these, the interviewed population prefers *cedro* (*Cedrela odorata*) and *coyo* (*Trichilia* sp.); however, these species have a low availability of individuals so their use is restricted. Thus, species growing on croplands such as *bili bil* (*Guarea guidonia*), *cachimbo* (*Erythrina fusca*), and *matarratón* (*Gliricidia sepium*) are often used instead.

Meanwhile, the *firewood* category grouped 30 species: *varazón* (*Casearia tremula*), *balso* (*Heliocarpus americanus*), *siete cueros* (*Machaerium* sp.), and *tachuelo* (*Zanthoxylum* sp.) are exclusive to this category. Other species of interest were *amargoso* (*Aspidodera cuspa*), *payandé* (*Pithecellobium dulce*), *cachimbo*, *matarratón*, *guácimo* (*Guazuma ulmifolia*), and *cuchiyuyo* (*Trichanthera gigantea*) (see Appendix). This category shares 13 species with fodder, which contains 28 species. In cropfields both cultivated (*leucaena* and *caña*) and wild species typical to tropical dry forests (*pela* and *amargoso*) are found.

The *medicine* category grouped the most species, 45, which are used for 32 different treatments. We found that a single species can be used for more than one treatment: such is the case of *sábila* (*Aloe vera*), *consuelda* (*Pseudelephantopus spiralis*), and *chisaca* (*Tridax procumbens*). The most used parts of the plant were twigs (52%) and leaves (26%). The most frequent forms of use were aromatic (57%) and poultice (15%), while the most frequent forms of application were oral (73%), external (15%), and in baths (6%) (Table 1).

**Table 1:** Ailment, form of preparation, and form of application of useful medicinal plants identified by inhabitats of the Doche vereda (Villavieja, Huila)

Common name	Scientific name	Used part	FP/FA	Ailment function
*Abreojo*	*Alternanthera* sp.	Tw	De/Or	Cough
*Aguacate*	*Persea americana* Mill.	Fr	De/Or	Circulation
*Albahaca*	*Ocimum basilicum* L.	Tw	To, De/Or, Ex	Tired eyes, diarrhea
*Amansa guapo*	*Justicia pectoralis* Jacq.	Le	Ar/Or	Relaxing
*Anamú*	*Petiveria alliacea* L.	Tw	De/Or	Ulcer, cancer
*Anón*	*Annona squamosa* L.	Le	De/Or	Vesicle
*Cacao*	*Theobroma cacao* (Mill.) Bernoulli	Fr	De/Or	Migraine
*Caléndula*	*Calendula* sp.	Tw	Ar/Or	Relaxing
*Chisacá*	*Tridax procumbens* L.	Tw	De/Or	Antipyretic, cough
*Cilantro cimarrón*	*Eryngium foetidum* L.	Tw	De/Or	Hepatitis
*Ciruelo*	*Spondias* sp.	Tw	Ju/Bt	Antipyretic, dysuria
*Consuelda*	*Pseudelephantopus spiralis* (Less.) Cronquist	Tw	Po, Ju/Ex	Anti-inflammatory, eye cleansing
*Cuchiyuyo*	*Trichanthera gigantea* (Bonpl.) Nees	Le	De/Bt, Or	Acne, cleansing the blood
*Desinchadera*	*Desmanthus virgatus* (L.) Willd.	Tw	Po/Ex	Anti-inflammatory
*Encaje*	N/I	Tw	De/Or	Dysuria
*Escoba*	*Sida rhombifolia* Mast.	Tw	De/Or	Diarrhea
*Gomo*	*Cordia dentata* Poir.	Fl	De/Or	Cough
*Guácimo*	*Guazuma ulmifolia* Pers.	Le	De/Or	Diarrhea
*Guanábano*	*Annona muricata* L.	Le	Po/Ex	Anti-inflammatory
*Guayaba cimarrón*	*Psidium guineense* Sw.	Fr	De/Or	Kidney
*Hita moreal*	N/I	St	Ju/Ex	Earache
*Insulina*	N/I	Tw	Ju, De/Ex, Or, Bt	Diabetes, kidney, abscesses
*Limón*	*Citrus × limón* (L.) Osbeck	Le	De/Or	Abdominal pain, diarrhea
*Limoncillo*	*Cymbopogon citratus* (DC.) Stapf	Tw	De, Ar/or	Abdominal pain, diarrhea, relaxing
*Mango*	*Mangifera indica* L.	Le	Po/Ex	Anti-inflammatory
*Marañón*	*Anacardium occidentale* L.	Tw	De/Or	Cough
*Matarratón*	*Gliricidia sepium* Kunth ex Steud.	Le	De/Or	Antipyretic
*Moringa*	*Moringa oleifera* Lam.	Le	Ar/Or	Relaxing, colon
*Mosquero*	*Croton pedicellatus* Kunth	Tw	De/Bt, Or	Diarrhea
*Naranjo*	*Citrus × sinensis* (L.) Osbeck	Le	De/Or	Accelerates labor
*Orégano*	*Origanum vulgare* L.	Le	De/Or	Tension
*Papaya*	*Carica papaya* L.	Fr	NP/Or	Colon, gastritis
*Pelá*	*Acacia farnesiana* Wall.	Ro, Ba	De/Bt, Or	Antipyretic
*Piñón*	*Jatropha curcas* (Adans.) Griseb.	Fr	NP/Or	Purging
*Pringamosa*	*Cnidoscolus urens* (L.) Arthur	Fl	Ar/Or	Cough
*Pronto alivio*	*Lippia alba* (Mill.) N.E. Br. ex Britton & P. Wilson	Tw	Ar/Or	Abdominal pain, relaxing
*Sábila*	*Aloe vera* (L.) Burm. f.	Le	De, Ju/Or	Prostate, diabetes; cough, gastritis, hair loss
*Simecojé*	*Momordica charantia* L.	Tw	De/Or	Gastritis, antipyretic
*Tomillo*	*Thymus* sp.	Tw	De/Or	Cold, cough
*Toronjil*	*Melissa* sp.	Tw	Ar/Or	Relaxing
*Verbena*	*Verbena litoralis* Kunth	Tw	Ju/Or	Antipyretic
*Verdolaga*	*Portulaca* sp.	Tw	De, Ju/Or	Dysuria, ulcer
*Yerbabuena*	*Mentha spicata* L.	Tw	Ar, De/Or	Abdominal pain, diarrhea, vomit
*Yuquillo*	N/I	Tw	De/Or	Cancer
*Sasafrás*	*Bursera tomentosa* (Jacq.) Triana & Planch.	Tw	De/Ex, Bt	Mosquito bites, rheumatism

Used part: *Ba* bark, *Fl* flower, *Fr* fruit, *Le* leaf, *Tw* twig, *Ro* root, *St* stalk. *FP* form of preparation: *Ar* aromatic, *De* decoction, *Po* poultice, *To* topical, *Ju* juice, *NP* no preparation. *FA* form of application: *Bt* baths, *Ex* external, *Or* oral, *N/I* not identified

In the *food* category, 20 species were registered. Of these, *piñuela* (*Bromelia penguin*) and *cabeza de negro* (*Melocactus curyispinus*) are not cultivated. Thirteen of these are also included in the *economy* category, among them are *yuca* (*Manihot esculenta*) and *plátano* (*Musa balbisiana*). Commerce of these species is carried out within the community of Doche vereda and in the municipality of Villavieja. The category *others* grouped 15 species, such as *nim (Azadirachta indica*), *gomo* (*Cordia dentate*), and *cruceto* (*Randia aculeate*). These species have diverse uses, such as insecticide, mouse poison, and home utensils. The category with the smallest amount of species in it is condiment, which includes five cultivated species (see Appendix).

Species with the greatest cultural significance according to their frequency of mention were *Pseudosamanea guachapele* (mentioned 18 times), *Guazuma ulmifolia* (17), *Manihot esculenta* and *Musa balbisiana* (16), and *Acacia farnesiana* and *Pithecellobium dulce* (15).

Species with the most uses were *Guazuma ulmifolia* and *Gliricidia sepium* each with five registered uses. Other multiple-use species are *Trichanthera gigantea*, *Annona muricata*, *Cordia dentata* and *Theobroma cacao*, each with four uses and, after these, 15 species with three uses, 26 species with two, and 53 species with only one registered use (see Appendix).

The results from the Pearson correlation coefficient found that there is a moderately positive relation (*r* = 0, 64; *p* < 0.001) between cultural importance and the number of ethnobotanical categories.

**Fig. 3:**
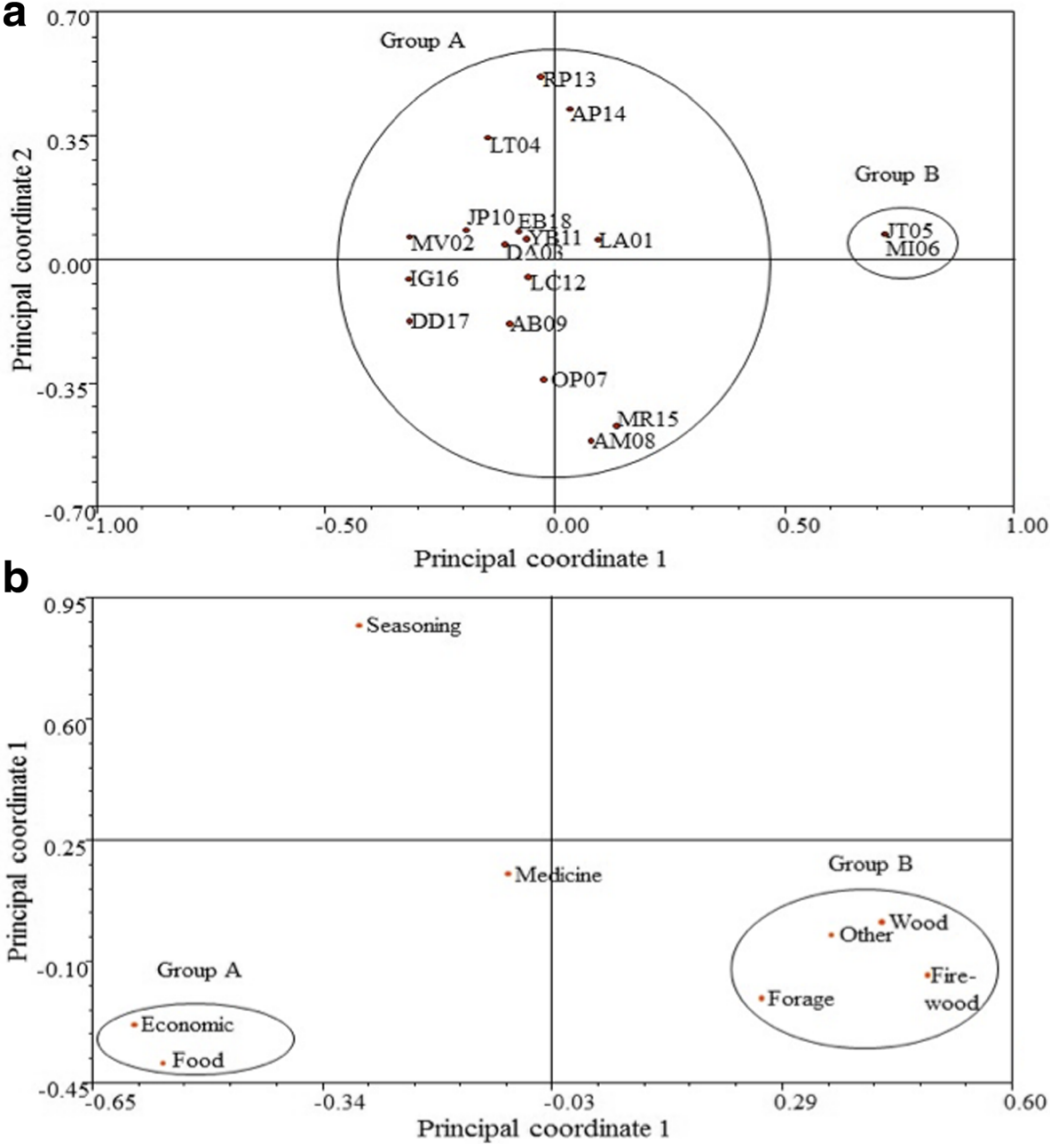
Principal Coordinates Analysis (PCO) **a** per interviewed person in the community of Doche vereda (Villavieja, Huila) and **b** per ethnobotanical category. Names of the interviewed population correspond to a code and an interview number

On the other hand, the Principal Coordinates Analysis (PCO) test made two interviewed groups. Most of the collaborators were in group A, while group B only included two interviewed persons (JT05, MI06) (Fig. 3). Furthermore, the PCO per ethnobotanical category also formed two groups (Fig. 3b); principal coordinate 1 discriminates the “timber”, “firewood”, “fodder”, and “others” grouping them apart from the “economic” and “food” categories.

The greatest Knowledge Richness (RQZ) was registered in three individuals (MI06, IG16, and JT05), each reaching a 0.35 value (Table 2). No significant differences between gender were found for RQZ values (*p* = 0, 6184). Similarly, no significant differences were found between genders within ethnobotanical categories (economy, *p* = 0.4011; fodder, *p* = 0.2152; firewood, *p* = 0.3813, timber, *p* = 0.4876; condiment, *p* = 0.1872; food, *p* = 0.3133; medicinal, *p* = 0.8904; and others, *p* = 0.5442).

**Table 2:** Number of uses per ethnobotanical category and Richness of Knowledge Index (RQZ) of the useful flora by interviewed of the Doche vereda (Villavieja, Huila)

Gender	Interviewed^a^	Fo	Co	Ec	Fd	Fi	Ti	Me	Ot	RQZ index
F	MI06	6	0	3	7	4	7	12	2	0.35
	IG16	7	1	4	7	7	2	15	1	0.35
	YB11	10	1	5	8	11	4	6	0	0.33
	OP07	6	1	3	8	7	3	6	1	0.27
	MV02	1	0	1	9	7	3	4	5	0.23
	AP14	2	0	2	6	7	4	5	4	0.23
	AM08	7	0	3	6	3	4	7	0	0.21
	LT04	0	0	0	6	4	5	6	2	0.18
	LC12	2	0	2	3	4	3	5	1	0.17
	AB09	3	0	3	4	5	2	2	0	0.16
	MR15	3	0	1	3	2	2	5	3	0.16
M	JT05	6	1	3	7	4	7	12	2	0.35
	JP10	3	1	3	7	11	10	7	0	0.25
	DA03	1	0	6	10	8	3	2	3	0.24
	DD17	4	1	7	6	7	2	6	0	0.24
	EB18	1	0	2	10	2	4	9	0	0.22
	RP13	2	0	2	8	8	2	6	2	0.21
	LA01	2	2	2	5	6	6	1	1	0.20

^a^Name and interview number abbreviated by interviewed individual

Ethnobotanical category: *Fo* food, *Co* condiment, *Ec* economy, *Fd* fodder, *Fi* firewood, *Ti* timber, *Me* medicine, *Ot* others

## Discussion

Of the useful flora in the Doche vereda, the family Fabaceae has the greatest diversity of genera and species, as has been formerly found in tropical dry forests of Huila [39, 52–54], in the Caribbean and in the dry valley of Magdalena river [55, 56]. Plants from this family are considered pioneers in the lowlands of the Neotropics, tropical dry forests, and arid and semi-arid zones [57, 58]. The use of legumes has physiological advantages by producing favorable habitats for the establishment of other species [59], their potential to become fodder, and being a relevant alternative in the management of areas and soil restoration [29, 52].

Regarding usage areas, the relevance of cropfields proved to be related to the coverage of immediate needs of the local population, by finding a greater availability and easy access to resources in these areas [49, 60]. Because of climate conditions in the area, farming these species guarantees resources that are relevant to the community for their role in covering basic needs. The relevance of cropland in peasant communities has been documented by Zuluaga and Ramírez [33], who found that these spaces contain and preserve a high agrodiversity, as well as accumulated knowledge that is a product of the experience of peasants adapting to production systems. These authors also reported 64 species in croplands, a similar number to that found in this work. Regarding the use of species within forests, a lower use to that reported for tropical dry forests of the Atlantic, Bolivar, Sucre, and Cesar [36, 43, 56]. This is explained by the subsistence needs of the Doche community, where cultivating species that guarantee certain services such as food, medicine, and fodder so that they are available yearlong is a priority.

When we analyzed by ethnobotanical category, we found 20 species within the *timber* category, which is less than what was reported in communities from the Complejo Ciénaga de Zapatosa and in Bailadores, Venezuela [34, 61], but similar to findings by Sanchez et al. [62], with 23 species of which only one (*Maclura tinctoria*) is cited in this work. The low availability of woody species resulting from the low rate at which this resource is produced in tropical dry forests, which is 50% slower than it is in tropical humid forests, has caused the substitution of these resources for cultivated species [63].

Quiroz-Carranza and Orellana [64] indicated the use of 41 species for firewood, which is more than we found in this study. The lower diversity of used species may be due to the preferences of inhabitants of the Doche community when selecting useful vegetation to use as firewood. The use of *leucaena*, *guanabano*, *guacimo*, and *payandé* is shared, since these species are easily grown in these dry ecosystems and have been reported to be preferred by peasant communities [65]. The recognition of firewood-bearing species can aid the preservation of tropical dry forests by pointing to strategies guaranteeing the availability of this resource and thus protect the species. The establishment of multiple use croplands close to the households [66], the extraction of dry timber from the forest in favor of woodcutting [64], and the design of stoves that increase the efficiency of firewood and keep spaces free of smoke are some of the suggested strategies to diminish the extraction of this resource from forests and allow its efficient use [67].

The *fodder* category grouped 28 species, more than what was reported by other studies [34, 35, 56, 68]. The number of species used as fodder in Doche is influenced by the cultivated plants and those collected from forests. Because of the climate conditions of the area, the community has implemented the cultivation of fodder species to avoid the displacement of cattle towards the higher areas of the forest. The use of *guacimo* [34], *cují* [62], *payandé*, and *leucaena* [69] has been previously reported as fodder species. Additionally, according to studies of the nutritional value of these plants to rumiants, the use of *matarratón*, *yuca*, and *leucaena* coincides with this study [70].

The *medicinal* category was the most important in the Doche community. When comparing the number of medicinal species reported in previous research in tropical dry forests, we found that the Atlantic, Bolivar, and Cesar have a lower use of species [35, 56], as is the case in the peasant community of Santa Catalina de Chongoyape in Peru [71]. Differences in the number of medicinal species are associated to resource availability, species uses, and the significance a plant has in the community [72].

The main ailments treated with these plants in the study area are epidemiologically frequent diseases in warm zones [73]. For the treatment of illness, those interviewed considered twigs to be the most effective, which coincides with research showing they contain a relatively high concentration of active substances and secondary metabolites [74, 75], particularly in the bark [76]. Although it is well known that other portions of plants contain a much larger concentration of metabolites, the use of the twigs instead of that of floral organs and of fruits is due to the fact that these organs present a low availability of the resource throughout the year as an adaptation strategy to the tropical dry forest [77, 78]. The use of twigs for medicinal use has already been documented in previous studies by Carrillo-Rosario and Moreno [79], Giraldo et al. [80], and Jaramillo et al. [81].

The *food* category grouped 20 species, which is less than what has been reported in other studies in tropical dry forests [34–36]. The use of tropical dry forest edible species in Ecuador was equally low (13 species), while in Mexico, Martínez-Pérez et al. [82] reported 51 species, far surpassing than what was found in this study. The apparently small number of used species might be due to the fact that all plants in this category are cultivated. Considering the climate conditions of the area and the need for this resource among its inhabitants, the community is focused on cultivating a small number of species in crop fields.

Out of 20 food species, 13 are also included in the *economy* category. These are sold in Doche and the populated center Villavieja. The sale of vegetable species generates an income that allows communities to obtain other basic needs [33], as well as projecting programs for the sustainable use of ecosystems, and through it, preservation strategies generate [83]. Such was the case of two communities in the Chietla municipality in Puebla, Mexico, where socio-economic and ecological valuation of the useful flora was generated in order to establish conservation priorities [82].

On the other hand, when we compared the plants grouped in the *others* category, we did not find coincidences with flora reported in other studies. Despite this fact, the relevance of shadow plants is widely recognized, and their multiple use as timber and fodder is recognized [34, 35].

Meanwhile, the *condiment* category reported the lowest number of species: five. The community does not utilize diverse spices to season foods, and all condiment plants are cultivated. This is also reported in communities from the Perijá Mountains where two species are registered, of these, *ají* coincides with our findings [35]. In the tropical region of Cesar, Colombia, the same number of species is reported and the use of *cilantro* (*Coriandrum sativum*) and *ají* (*Capsicum annuum*) coincides with this study [34].

According to the results from the Pearson correlation test, a significant relationship was found between the number of uses per ethnobotanical category and cultural significance (see Appendix). Thus, species with the highest cultural significance will be those with the most different uses. To some authors, the frequency of being mentioned is a very effective indicator for the evaluation of cultural importance, mainly because it is a quick technique and relatively easy to carry out; however it does not say much about the particular importance of the different species [84]. On the other hand, literature points out that as long as a plant has more uses (food, medicine, fuel, or any other category), it will have greater importance [21, 22]. Different ethnobotanical studies have indicated that the sum of uses can be considered as an indicator that is directly related to the cultural importance of the plants and that the sum of uses can be a quick tool that provides quantitative data to evaluate this phenomenon [16]. Thus, based on the evidence, we can see how the number of uses of a plant is correlated with its degree of cultural importance. This allows us to recognize the relevance of these species and opens the possibility to generate management and preservation strategies in different ecosystems in future studies [85]. Additionally, it would be recommendable to use standardized ethnobotanical categories to compare between studies and study sites [86].

On the other hand, the Principal Coordinates Analysis per ethnobotanical category, showed the relationship between species and ethnobotanical categories. The first Principal Coordinate grouped species considered edible for the community that also provide families with income (“food” + “economy”; see Fig. 3b). Meanwhile, the second Principal Coordinate discriminated a greater number of categories, all of which share similar uses and are recognized by most of the interviewed population, such as timber species that can also be fodder for goats and sheep. According to this, a single species may be used in more than one category. This has been made reference to before in the study by Cárdenas and Ramírez [29]; however, to avoid bias in value allocation, it is recommended to cite the species once per category instead of according to each use given. This was done in this study according to proposals by Marín-Corba et al. [86] and Sánchez et al. [87].

The degree of knowledge of the useful species by collaborators in the Doche community, as measured through the Knowledge Richness Index, has no significant differences between genders. According to different authors, there are differences between men and women in knowledge about natural resources [27]. For Tuñón [88], the differences between the uses, access, and control that men and women have of their natural resources are evident. It can even be expected that women hold greater knowledge. To Sánchez-Núñez and Espinosa [89], women have a detailed knowledge about the natural environment that places them in a preponderant place in the administration of community natural resources. However, in the present study there seems to be no gender difference in the richness of knowledge. Although the level of plant knowledge varies, each person has a portion of the “total” knowledge and it can change according to necessities and priorities in each community as is concluded by Castellanos [23] in his study of the Cane river basin in Iguaque (Boyacá). Predominant economic activities, urbanization, individual roles, and cultural diversity are among the factors influencing how much communities know of their ecosystems and how they use them [61, 90].

Similarly, no significant differences were found between genders within ethnobotanical categories. Communities in greater contact with their ecosystems tend to relate the same species [85]. Differences in knowledge and perception of natural resources between men and women have been partially explained as a consequence of the sexual division of labor in traditional societies [27]. Nevertheless, in the Doche community, both men and women carry out similar activities, which can be evidenced in this study by their reporting the same useful species. This coincides with reports from Canales et al. [91] who indicated that the number of known plants is not related to the area they inhabit nor to gender, schooling, occupation, or place of origin, but rather to the role each person plays in a community and the activities they carry out in it. However, certain tendencies can be observed in some categories, for example, medicinal species are more readily recognized by women [26, 46], as are food species [92], while men have deeper knowledge of species used in construction and sold species [25]. Voeks and Leony [93] report that women from a rural community in the state of Bahia, Brazil, are significantly better informed than men about the names and medicinal properties of plants. Although our data did not support the hypothesis that there is a difference in the degree of knowledge between genders, within particular categories, such as medicinal plants, we found evidence that this difference does appear. According to the results of the PCO per category, a consensus was found in the information about useful plants, which would suggest homogeneity and preservation of traditional knowledge, contrasting with findings by Albuquerque et al. [13] and Lastres et al. [60], who cite traditional knowledge to be disperse among the people, which might lead to its eventual loss.

Finally, the relevance of culturally significant species has led to the recognition of resource availability and the knowledge communities have of plants [81, 94]. Additionally, strategies for a better use of ecosystems can be put into practice considering the most relevant species [34]. Cultural valuation should identify, recognize, and accept changes in preferences and the dynamic way in which communities learn, given that people are constantly modifying their ecosystems in search for optimal benefits [95]. This is evident in Doche, a community that has modified its agricultural and livestock-breeding practices to guarantee a long-term availability of the resources their environment provides. Furthermore, people in this community have received training to generate new strategies to utilize their ecosystems, as well as regulate the use or resources such as the wood that is extracted from the forest.

## Conclusions

The usage strategies developed by people from Doche to thrive in their dry ecosystem lead us to the conclusion that knowledge of the cultural value of vegetable species is fundamental to endeavor in forest preservation without ceasing to use natural resources. The agreements on internal rules to control the extraction of timber and the cultivation of species for this purpose are strategies that this community have developed to preserve resources while still covering their basic needs. The establishment of cultivation crop lands, the limitation of livestock breeding within the forests, and the diversification of species has further contributed to the regeneration of the tropical dry forest.

The cultural valuation measured in the frequency of mention allowed us to recognize the cultural significance of species of the tropical dry forest; however, the importance of these species was explained by diverse factors such as the number of uses per ethnobotanical category, availability, and access to the resource. Furthermore, the recognition of ethnobotanical uses by gender showed that in the Doche community, men and women know the same species, both genders participate in agricultural endeavors, the collection of wood, herding, production of food, and selling, as well as group activities carried out in the community. This has led to a homogeneously distributed and well-preserved ethnobotanical knowledge.

The community is more invested in preserving species with a higher cultural significance because these provide them with basic resources for subsistence. Each person has a portion of the general knowledge, and this is modified according to immediate needs, as well as the availability and access to resources. Therein lies the relevance of recognizing useful species, use areas, and socio-ecological relationships between a population and its ecosystem. The knowledge of useful flora and its cultural valuation represents a relevant step towards the preservation of the tropical dry forest, one of the most fragmented ecosystems in Colombia. The participation of communities in the preservation of this ecosystem is fundamental for strategies to guarantee a long-term conservation of this ecosystem and the services with which it provides the population.

## Appendix

**Table 3:** Species used by the Doche commnity (Villavieja, Huila)

Useful Flora	Common name	Zone	Ethnobotanical category	Total Uses	Freq. of being mentioned	Relative freq. of being mentioned
Achariaceae						
*Carpotroche grandiflora* Spruce ex Benth.	*Achotillo*	F	Ot	1	2	0.11
*Justicia pectoralis* Jacq.	*Amansa guapo*	C	Me	1	1	0.06
*Trichanthera gigantea* (Bonpl.) Nees	*Cuchiyuyo*	C	Ti; Fi; Ff; Me	4	4	0.22
Amaranthaceae						
*Alternanthera* sp.	*Abreojo*	F; C	Me	1	5	0.28
Anacardiaceae						
*Anacardium occidentale* L.	*Marañón*	C	Me	1	1	0.06
*Astronium graveolens* Jacq.	*Diomate*	F	Ti	1	4	0.22
*Mangifera indica* L.	*Mango*	C	Fo; Me	2	2	0.11
*Spondias* sp.	*Ciruelo*	C	Me	1	4	0.22
Annonaceae						
*Annona muricata* L.	*Guanábano*	C	Fi; Fo; Me; Ot	4	3	0.17
*Annona squamosa* L.	*Anón*	C	Me	1	1	0.06
Apiaceae						
*Coriandrum sativum* L.	*Cilantro*	C	Co; Ec	2	1	0.06
*Eryngium foetidum* L.	*Cilantro cimarrón*	C	Co; Me	2	3	0.17
Apocynaceae						
*Aspidosperma cuspa* (Kunth) S.F. Blake ex Pittier	*Amargoso*	F	Ti; Fi; Fd	3	10	0.56
Araceae						
*Xanthosoma sagittifolium* (L.) Schott	*Bore*	C	Fd	1	5	0.28
Arecaceae						
*Bactris gasipaes* Kunth	*Cachipay; Chontaduro*	C	Fo	1	1	0.06
*Cocos nucifera* L.	*Coco*	C	Fo	1	1	0.06
Asphodeloideae						
*Aloe vera* (L.) Burm. f.	*Sábila*	C	Me; Ec	2	7	0.39
Asteraceae						
*Calendula* sp.	*Caléndula*	C	Me	1	1	0.06
*Lactuca sativa* L.	*Lechuga*	C	Fo; Ec	2	1	0.06
*Pseudelephantopus spiralis* (Less.) Cronquist	*Consuelda; yerbagole; hierba golpe*	C	Fd; Me	2	2	0.11
*Tridax procumbens* L.	*Chisacá*	C	Me	1	8	0.44
Boraginaceae						
*Cordia dentata* Poir.	*Gomo*	C	Fi; Fd; Me; Ot	4	6	0.33
Bromeliaceae						
*Bromelia pinguin* L.	*Piñuela*	F	Fo	1	1	0.06
Burseraceae						
*Bursera graveolens* (Kunth) Triana & Planch.	*Tatamaco*	F	Ti	3	5	0.28
*Bursera tomentosa* (Jacq.) Triana & Planch.	*Zazafra; sasafrás*	F	Ti; Fi; Me	1	2	0.11
Cactaceae						
*Melocactus curvispinus* Pfeiff.	*Cabeza de negro*	F	Fo	1	3	0.17
*Stenocereus griseus* (Haw.) Buxb.	*Cardo*	F	Fi; Fd	2	9	0.50
Caricaceae						
*Carica papaya* L.	*Papaya*	C	Fo; Me	2	4	0.22
Convolvulaceae						
*Ipomoea* sp.	*Calambrino*	F	Fd	1	1	0.06
Cucurbitaceae						
*Momordica charantia* L.	*Simecojé*	C	Me	1	1	0.06
Euphorbiaceae						
*Cnidoscolus urens* (L.) Arthur	*Pringamosa*	F	Me	1	5	0.28
*Croton pedicellatus* Kunth	*Sanca de arriero; Mosquero*	F	Fd; Me; Ot	3	9	0.50
*Jatropha curcas* L.	*Piñón*	C	Me	1	1	0.06
*Manihot esculenta* Crantz	*Yuca*	C	Fd; Fo; Ot	3	16	0.89
*Pedilanthus tithymaloides* (L.) Poit.	S/N	F	Fd	1	1	0.06
Lamiaceae						
*Melissa officinalis* L.	*Toronjil*	C	Me	1	2	0.11
*Mentha spicata* L.	*Yerbabuena*	C	Co; Me	2	9	0.50
*Ocimum basilicum* L.	*Albahaca*	C	Me	1	2	0.11
*Origanum vulgare* L.	*Orégano*	C	Co; Me	2	5	0.28
*Thymus* sp.	*Tomillo*	C	Me	1	2	0.11
Lauraceae						
*Persea americana* Mill*.*	*Aguacate*	C	Fo; Me	2	1	0.06
Fabaceae						
*Acacia farnesiana* (L.) Willd.	*Pelá*	F	Fi; Fd; Me	3	15	0.83
*Acacia* sp.	*Ambuco*	F	Ti; Fi; Fd	3	10	0.56
*Desmanthus virgatus* (L.) Willd.	*Desinchadera*	F; C	Fd; Me	2	6	0.33
*Erythrina fusca* Lour.	*Cachimbo*	C	Ti; Fi	2	2	0.11
*Gliricidia sepium* (Jacq.) Kunth ex Walp.	*Matarratón*	C	Ti; Fi; Fd; Me; Ot	5	13	0.72
*Hymenaea courbaril* L.	*Algarrobo*	F; C	Fi; Fd; Ot	3	6	0.33
*Leucaena leucocephala* (Lam.) de Wit	*Leucaena*	C	Ti; Fi; Fo	3	10	0.56
*Machaerium* sp.	*Siete Cueros*	F	Fi	1	1	0.06
*Parkinsonia aculeata* L.	*Retamo*	F	Fd	1	1	0.06
*Pithecellobium* sp.	*Mulato*	F	Ti; Fi	2	2	0.11
*Pithecellobium dulce* (Roxb.) Benth.	*Payandé*	F	Ti; Fi; Fd	3	15	0.83
*Prosopis juliflora*	*Cují*	F	Fi; Fd; Ot	3	9	0.50
*Pseudosamanea guachapele* (Kunth) Harms	*Iguá*	F; C	Ti; Fi	2	18	1.00
*Senna* sp.	*Chilinchil*	F	Fi; Ot	2	2	0.11
*Tamarindus indica* L.	*Tamarindo*	C	Fo	1	1	0.06
Loranthaceae						
*Oryctanthus* sp.	*Pajarito*	F; C	Fd	1	2	0,11
Malvaceae						
*Gossypium* sp.	*Algodón*	C	Ec	1	1	0.06
*Guazuma ulmifolia* Lam.	*Guácimo*	F; C	Ti; Fi; Fd; Me; Ec	5	17	0.94
*Heliocarpus americanus* L.	*Balso*	F; C	Fi	1	1	0.06
*Sida rhombifolia* L.	*Escoba*	C	Me; Ot	2	2	0.11
*Theobroma cacao* L.	*Cacao*	C	Fi; Fo; Me; Ec	4	12	0.67
Meliaceae						
*Azadirachta indica* A. Juss.	*Nim*	C	Ot	1	1	0.06
*Cedrela odorata* L.	*Cedro*	F	Ti	1	4	0.22
*Guarea guidonia* (L.) Sleumer	*Bili bil*	C	Ti	1	6	0.33
*Trichilia appendiculata* (Triana & Planch.) C. DC.	*Yayo*	F	Ti	1	3	0.17
*Trichilia* sp.	*Coyo*	F	Ti	1	4	0.22
Moraceae						
*Artocarpus altilis* (Parkinson) Fosberg	*Juan Pana*	C	Fd; Fo	2	2	0.11
*Maclura tinctoria* (L.) D. Don ex Steud.	*Dinde*	F	Ti: Fd	2	9	0.50
Moringaceae						
*Moringa oleífera* Lam.	*Moringa*	C	Fd; Me; Ot	3	3	0.17
Muntingiaceae						
*Muntingia calabura* L.	*Chicható*	F; C	Fi; Fo; Ot	3	3	0.17
Musaceae						
*Musa balbisiana* Colla	*Plátano*	C	Fo; Al; Ot	3	16	0.89
Myrtaceae						
*Myrcia* sp.	*Arrayán*	F	Ti	1	1	0.06
*Psidium guajava* L.	*Guayaba*	C	Fo	1	1	0.06
*Psidium guineense* Sw.	*Guayaba cimarrón*	F	Me	1	1	0.06
Phytolaccaceae						
*Petiveria alliacea* L.	*Anamú*	C	Me	1	3	0.17
Poaceae						
*Brachiaria decumbens* Stapf	*Pasto*	C	Fd; Ec	2	13	0.72
*Cymbopogon citratus* (DC.) Stapf	*Limoncillo*	C	Me	1	6	0.33
*Guadua angustifolia* Kunth	*Guadua*	C	Fi; Ot	2	8	0.44
*Gynerium sagittatum* (Aubl.) P. Beauv.	*Pinto; pindo*	C	Fd; Ot	2	3	0.17
*Oryza sativa* L.	*Arroz*	C	Fo; Ec	2	3	0.17
*Saccharum officinarum* L.	*Caña*	C	Fi; Fo	2	4	0.22
*Sorghum bicolor* (L.) Moench	*Sorgo*	C	Ec	1	1	0.06
*Zea mays* L.	*Maíz*	C	Fd; Fo; Ec	3	8	0.44
Portulacaceae						
*Portulaca* sp.	*Verdolaga*	F	Me	1	3	0.17
Rubiaceae						
*Machaonia acuminata* Bonpl.	*Cacho de venado*	F	Fi	1	5	0.28
*Randia aculeata* L.	*Cruceto*	F	Fi; Ot	2	3	0.17
Rutaceae						
*Amyris pinnata* Kunth	*Milanda*	F	Ti	1	1	0.06
*Citrus × sinensis* (L.) Osbeck	*Naranjo*	C	Fo; Me	2	2	0.11
*Citrus × limón* (L.) Osbeck	*Limón*	C	Fo; Me; Ec	3	6	0.33
*Zanthoxylum* sp.	*Tachuelo*	F	Fi	1	1	0.06
Salicaceae						
*Casearia tremula* (Griseb.) Griseb. ex C. Wright	*Varazón*	F	Fi	1	1	0.06
*Sideroxylon celastrinum* (Kunth) T.D. Penn.	*Chisposo*	F	Fd	1	1	0.06
Solanaceae						
*Capsicum annuum* L.	*Ají*	P	Co	1	2	0.11
Verbenaceae						
*Lippia alba* (Mill.) N.E. Br. ex Britton & P. Wilson	*Pronto alivio*	C	Me	1	3	0.17
*Verbena litoralis* Kunth	*Verbena*	C	Me	1	1	0.06
Not identified						
Sp 1.	*Encaje*	C	Me	1	2	0.11
Sp 2.	*Hita moreal*	C	Me	1	1	0.06
Sp 3.	*Insulina*	C	Me	1	1	0.06
Sp 4.	*Yuquillo*	F	Me	1	1	0.06

Species use zone: *C* cropland, *F* forest. Ethnobotanical category: *Fo* food, *Co* condiment, *Ec* economy, *Fd* fodder, *Fi* firewood, *Ti* timber, *Me* medicine, *Ot* others, *S/N* no common name

## Abbreviations

Ar: Aromatic
Ba: Bark
Bt: Baths
C: Cropland
Co: Condiment
De: Decoration
Ec: Economy
Ex: External
F: Forest
FA: Form of application
Fd: Fodder
Fi: Firewood
Fl: Flower
Fo: Food
FP: Form of preparation
Fr: Fruit
Ju: Juice
Le: Leaf
Me: Medicine
N/I: Not identified
NP: No preparation
Or: Oral
Ot: Others
PCO: Principal Coordinates Analysis
Po: Poultice
Ro: Root
RQZ: Knowledge Richness Index
S/N: No common name
St: Stalk
Ti: Timber
To: Topical
Tw: Twig
